# Hippocampal Neural Stem Cell Grafting after Status Epilepticus Alleviates Chronic Epilepsy and Abnormal Plasticity, and Maintains Better Memory and Mood Function

**DOI:** 10.14336/AD.2020.1020

**Published:** 2020-12-01

**Authors:** Bharathi Hattiangady, Ramkumar Kuruba, Bing Shuai, Remedios Grier, Ashok K Shetty

**Affiliations:** ^1^Institute for Regenerative Medicine, Department of Molecular and Cellular Medicine, Texas A&M University College of Medicine, College Station, TX, USA.; ^2^Research Service, Olin E. Teague Veterans' Medical Center, Central Texas Veterans Health Care System, Temple, TX, USA.; ^3^Department of Surgery (Neurosurgery) Duke University Medical Center, Durham, NC, USA.; ^4^Research and Surgery Services, Durham Veterans Affairs Medical Center, Durham, NC, USA

**Keywords:** cell transplantation, cognitive dysfunction, depression, EEG, hippocampal NSCs, memory, neural stem cells, neuroprotection, stem cell grafts, temporal lobe epilepsy

## Abstract

Hippocampal damage after status epilepticus (SE) leads to multiple epileptogenic changes, which lead to chronic temporal lobe epilepsy (TLE). Morbidities such as spontaneous recurrent seizures (SRS) and memory and mood impairments are seen in a significant fraction of SE survivors despite the administration of antiepileptic drugs after SE. We examined the efficacy of bilateral intra-hippocampal grafting of neural stem/progenitor cells (NSCs) derived from the embryonic day 19 rat hippocampi, six days after SE for restraining SE-induced SRS, memory, and mood impairments in the chronic phase. Grafting of NSCs curtailed the progression of SRS at 3-5 months post-SE and reduced the frequency and severity of SRS activity when examined at eight months post-SE. Reduced SRS activity was also associated with improved memory function. Graft-derived cells migrated into different hippocampal cell layers, differentiated into GABA-ergic interneurons, astrocytes, and oligodendrocytes. Significant percentages of graft-derived cells also expressed beneficial neurotrophic factors such as the fibroblast growth factor-2, brain-derived neurotrophic factor, insulin-like growth factor-1 and glial cell line-derived neurotrophic factor. NSC grafting protected neuropeptide Y- and parvalbumin-positive host interneurons, diminished the abnormal migration of newly born neurons, and rescued the reelin+ interneurons in the dentate gyrus. Besides, grafting led to the maintenance of a higher level of normal neurogenesis in the chronic phase after SE and diminished aberrant mossy fiber sprouting in the dentate gyrus. Thus, intrahippocampal grafting of hippocampal NSCs shortly after SE considerably curbed the progression of epileptogenic processes and SRS, which eventually resulted in less severe chronic epilepsy devoid of significant cognitive and mood impairments.

Chronic temporal lobe epilepsy (TLE) is characterized by periodic and unpredictable occurrences of partial complex seizures and variable levels of impairments in learning, memory, and mood [1-5]. Although the cause is unspecified in most instances, TLE is a result of an initial precipitating injury (IPI) such as status epilepticus (SE), stroke, head trauma, encephalitis, or childhood febrile seizures in other cases [6-7]. For example, damage to the hippocampus induced by SE leads to multiple epileptogenic changes, which eventually evolve into chronic TLE [8-14]. Chronic TLE development after an IPI may involve a latent period of weeks, years, or even decades. Administration of a combination of antiepileptic drugs (AEDs) after an IPI is efficient for terminating acute seizures in most cases. However, AED therapy cannot thwart the various epileptogenic changes that ensue after an IPI [15-17]. Therefore, the development of alternative therapies that are efficient for blocking or reducing the progression of IPI-induced epileptogenic changes has considerable significance for positively modulating hippocampal plasticity after injury [18-20]. If efficient, such therapies would likely prevent or delay the onset of TLE or at least reduce TLE's intensity after an IPI.

Neural stem/progenitor cell grafting into the damaged hippocampus has promise for restraining epileptogenesis after an IPI because of multiple reasons. After grafting, NSCs can survive, proliferate at least a few times, and migrate to different layers of the damaged hippocampus [21]. The grafted NSCs give rise to three major cell types in the brain (neurons, astrocytes, and oligodendrocytes) even when faced with an adverse microenvironment [21-22]. Moreover, a fraction of neurons produced by NSCs differentiates into gamma-aminobutyric acid (GABA) producing interneurons, which may partially replenish SE-induced loss of host GABA-ergic interneurons [21-23]. Furthermore, NSCs can mediate neuroprotective effects through the release of multiple beneficial neurotrophic factors such as the brain-derived neurotrophic factor (BDNF), glial cell line-derived neurotrophic factor (GDNF), insulin-like growth factor (IGF-1), and fibroblast growth factor-2 (FGF-2) [21-24]. Besides, after a unilateral hippocampal injury, NSC grafting can prevent cognitive, memory, and mood impairments [21]. Also, if found efficient for restraining epilepsy development in animal models, NSC grafting can be translated to the clinic because such cells can be obtained from multiple sources. These include post-mortem fetal, postnatal, or adult brain tissues, human embryonic stem cells, human induced pluripotent stem cells, and induced NSCs obtained through direct conversion of human somatic cells [23, 25-29]. More importantly, NSCs are amenable for expansion and characterization in culture for obtaining a desirable number and type of cells for clinical application [29].

In this study, we examined the efficacy of intrahippocampal grafting of the rat fetal hippocampus-derived NSCs after SE for restraining several significant epileptogenic changes, SRS, and memory and mood impairments. The donor NSCs were expanded in culture as neurospheres from the embryonic day 19 (E19) rat hippocampi, labeled with 5'-Chloro-2'-deoxyuridine (CldU) and treated with BDNF before grafting. Grafting was performed bilaterally into hippocampi six days after an SE induced through graded injections of kainic acid (KA) [9, 10, 22, 30]. Grafted animals were measured for the frequency, intensity, and duration of behavioral SRS through direct observations at 3-5 months after SE, in comparison to age-matched animals subjected to SE alone and animals receiving SE and dead cell grafts. Next, at six months after SE, grafted animals were examined for memory and depressive-like behavior compared to animals subjected to SE alone and naïve control animals. Following this, subsets of animals from both SE alone and grafted groups were measured for the frequency, intensity, and duration of SRS using continuous video-encephalographic (video-EEG) recordings at eight months post-SE. Animals were euthanized nine months after SE to measure graft cell yield, migration, and differentiation, and long-term effects of grafts on the survival of host interneurons expressing neuropeptide Y (NPY), parvalbumin (PV) and reelin, aberrant migration of newly born neurons, the extent and pattern of ongoing neurogenesis and anomalous mossy fiber sprouting in the dentate gyrus (DG).

## MATERIALS AND METHODS

The sequence and timeline of various experiments, and data collection and analyses, are illustrated in a flowchart (Supplementary Fig. 1).

### Experimental groups and grafting of NSCs

Young adult (5-months old) male Fischer 344 rats (Harlan Sprague-Dawley, Indianapolis, IN) were treated with graded intraperitoneal injections of Kainic acid (KA; 3.0 mg/kg/h) to induce SE, as detailed in previous reports [8, 10, 31, 32]. The motor seizures during SE were scored as per the modified Racine scale [8], and the behavioral seizures were terminated 2 hours after SE onset through a diazepam injection (5mg/kg). Animal studies were accomplished in accordance with the NIH guidelines for care and use of animals and animal protocols approved by the animal care and use committees (IACUCs) of the VA Medical Centers (Durham, NC, and Temple, TX), Duke University Medical Center, and the Texas A&M Health Science Center. We employed only male rats in this study because the induction of SE, survival after SE, epileptogenic changes, and chronic epilepsy development have been well characterized for this gender in our previous studies [8, 10, 31, 32].

### Harvesting, expansion, and CldU labeling of hippocampal NSCs

NSCs from the embryonic day 19 (E19) hippocampi were expanded, as detailed in our previous publication [33]. Embryonic day 19 (E19) fetuses were removed from deeply anesthetized timed pregnant F344 rats by cesarean section, and heads collected in a proliferation medium containing Dulbecco's modified Eagle's Medium (DMEM)/F12 (3:1 mixture; Life Technologies, Gaithersburg, MD), B-27 supplement (1ml/50 ml of the medium; Life Technologies), EGF (20 ng/ml; Sigma), FGF-2 (20 ng/ml; Peprotech), heparin (5 mg/ml), penicillin (100U/ml; Sigma), and streptomycin (100g/ml; Sigma). The brains were next dissected out in a Petri plate containing the same proliferation medium, and the hippocampus was isolated under a Nikon dissection microscope, triturated and processed, and plated as detailed in our previous publication [33]. The next day, the proliferating cells were labeled by adding 3 µM of 5'-Chloro-2'-deoxyuridine (CldU; an analog of 5'-bromodeoxyuridine) into the culture medium and maintained until day 6. On day 7, the neurospheres were collected, washed, and mechanically dissociated to get a suspension of single cells and small clusters. The cells were washed twice in a fresh culture medium, centrifuged, viability assessed through a trypan blue exclusion test. The viable cells were adjusted to a density of 80,000 cells/µl, and the final cell suspension was treated with the brain-derived neurotrophic factor (BDNF, 200 ng/ml) before grafting. Only cell suspensions that exhibited ≥75% viability were used for grafting. CldU labeling index was determined by CldU immunostaining after overnight incubation of dissociated cells on poly-L-lysine-coated culture dishes containing differentiation medium comprising Neurobasal medium (95.5ml/100ml), B-27 (2ml/100ml), L-glutamine (1.25ml/100ml), penicillin (100U/ml) and streptomycin (100ug/ml).

### Animals, induction of SE and NSC grafting

The study comprised four groups of animals: age-matched naïve control group (n=9), a group receiving no treatment after SE (SE only group, n=10), a group receiving hippocampal NSC grafts after SE (SE+NSC group, n=8), and animals receiving dead hippocampal NSCs after SE (SE+DC group, n=6). Animals in the SE + NSC group received bilateral grafting of NSCs into the hippocampus (80,000 live cells/site, 4 sites/each hippocampus), 6 days after SE, whereas animals in the SE + DC group received the same amount of dead hippocampal NSCs. The donor NSCs were killed by repeated freezing and thawing, and the death of virtually all cells in the suspension was confirmed through the trypan blue test. The following stereotaxic coordinates were employed for grafting: (i) anteroposterior (AP) = 3.0 mm from bregma, lateral (L) = 1.8 mm from midline, and ventral (V) = 3.5 mm from the surface of the brain; (ii) AP = 3.6 mm, L = 2.5 mm, V = 3.5 mm; (iii) AP = 4.2 mm, L = 3.0 mm, V = 3.5 mm; (iv) AP = 4.8 mm, L = 3.5 mm, V = 4.0 mm. Additional details on the grafting procedure are described in our previous publications [11, 31, 34]. Since our previous studies have demonstrated that allogeneic neural cell grafts could survive for prolonged periods without immune suppression [9, 11, 21, 23], immunosuppression was not performed in this study.

### Measurement of behavioral SRS

The identity of animals in epileptic groups (SE alone, SE+NSC, and SE+DC) were blinded through experimental codes, and the extent of behavioral SRS was measured for three months commencing at three months after grafting (two 4-hour sessions/week, 32 hours/month) as described previously [8]. The SRS measurements comprised the average frequency of all and stage-V SRS, the average duration of individual SRS, and the total time spent in seizure activity for the recording period. Cumulative seizure scores were next compared between groups for every month using repeated-measures ANOVA.

### Behavioral tests for measuring memory and mood function

Animals in naïve, SE alone, and SE+NSC groups were examined with specific behavioral tests that measured recognition memory and mood function, as described in previous reports [21, 35-40]. In epileptic groups (SE alone and SE+NSC), the behavioral tests were done at ~6 months post-SE. First, we employed a novel object recognition test (NORT) to measure the recognition memory function. In this test, rats were examined for their ability to discriminate a novel object from a familiar object using an open filed apparatus. A higher propensity to explore the novel object than the familiar object in the memory testing phase implies the use of learning and recognition memory processes. A detailed portrayal of this test is available in our earlier publications [21, 35-37]. Following NORT, we examined mood function in animals belonging to different groups using a forced swim test (FST), which is one of the widely used behavioral paradigms for measuring depressive-like behavior (or learned helplessness) in rodents [38, 39]. In this test, each rat was forced to swim for 10 minutes in an upright cylinder filled two-thirds with water. The extent of depressive-like behavior is directly proportional to the amount of time spent in floating. Additional details on this test are available in our earlier reports [21, 35, 40].

### Measurement of video-encephalographic (video-EEG) recordings

Since the extent of behavioral SRS was similar between SE only and SE+DC groups, continuous video-EEG recordings were measured only from SE only and SE+NSC groups. For these recordings, subgroups of animals from SE only and SE+NSC groups underwent EEG implantation surgery at eight months after SE. Video-EEG traces were continuously recorded from a surface electrode placed on the frontoparietal cortex for ~108 hours (AS40: Grass Telefactor). The EEG implantation surgery and EEG recordings were done as described in our previous reports [8, 41]. A time-locked video-EEG monitoring system (AS40 from Grass Telefactor) was employed. Each rat was anesthetized and fixed to a stereotactic device. Burr holes were made in the skull to implant EEG recording electrodes and screws. Three sterile metal EEG recording electrodes with mounting screws (Plastics One) were placed epidurally over the right frontoparietal cortex (recording electrode#1), the left frontoparietal cortex (recording electrode #2), and the left cerebellum (reference electrode), respectively. Besides, 1-2 anchoring screws without electrodes were placed over the frontal cortex. The screws and electrodes were cemented in place, and electrode leads were attached to a micro-plug and then cemented to the animal’s head. Two weeks later, each rat was placed in a Plexiglas cage, the connector cable of the video-EEG system was fixed into the electrode pedestal on the rat’s head. The video-EEG system monitored simultaneously occurring behavior and EEG activity in awake, freely behaving rats with ad libitum access to food and water. The EEG recordings were done continuously for 108 hours with low-frequency filter (LF) set at 0.3Hz, high frequency (HF) set at 35 Hz, and data rate at 200 Hz. The EEG tracings were analyzed for the frequency of all SRS, the frequency of stage V-SRS (the most severe form of SRS), the average duration of individual SRS, and the total time spent in seizure activity for the recording period.

### Tissue processing

After the completion of behavioral tests and EEG recordings, animals in all groups were perfused. In epileptic groups, the timing of perfusion was equivalent ~9 months after SE. Each animal was transcardially perfused with 4% paraformaldehyde, following which the brain was dissected and post-fixed in 4% paraformaldehyde for ~18 hours and processed for histology. Thirty micrometer thick coronal sections were cut through the entire hippocampus using a cryostat and collected serially in 24-well plates containing phosphate buffer.

### Analyses of the yield, differentiation of graft-derived cells

In every group, a set of serial sections (every 15th) through the entire hippocampus was processed for neuron-specific nuclear antigen (NeuN) immunostaining [31] to examine neurodegeneration. Another set of serial sections (every 10th) from grafted animals was processed for CldU immunostaining using a rat anti-BrdU antibody (1:300; Serotech: Raleigh) [22, 31]. The cells positive for CldU were then counted using a Stereo Investigator system (Microbrightfield). The stereological counting procedure employed is detailed in our previous reports [9, 21-23]. The yield of transplant-derived cells in each hippocampus was expressed as the percentage of injected CldU+ cells (i.e., 320,000 live cells through 4 grafts). We characterized the phenotype of graft-derived cells through dual immunofluorescence for CldU with markers of various neural cell antigens. These include the mature neuronal marker NeuN (mouse anti-NeuN, 1:1000; Millipore), an inhibitory interneuron marker GABA (rabbit anti-GABA, 1:5000; Sigma-Aldrich), a mature astrocyte marker S-100β (mouse anti-S100β, 1:1000; Millipore), an oligodendrocyte marker O4 (mouse anti-O4: 1:500, R&D Systems), and an oligodendrocyte progenitor cell marker NG2 (Rabbit anti-NG2, 1: 500; Millipore). Dual-labeled cells were examined and quantified using Z-section analyses in a confocal microscope (FV10i, Olympus). The presence of dual-labeling was confirmed when any neural antigen was co-expressed in 4-5 serial Z-sections taken at one-micrometer intervals. For the quantification of each neural cell antigen, 100-150 graft-derived cells were examined from the grafted hippocampus (n=4-5).

### Measurement of neurotrophic factor expression in graft-derived cells

The hippocampal sections containing grafts were processed for dual immunofluorescence staining using anti-CldU antibody and specific antibodies against neurotrophic factors BDNF (1: 1000, SCBT), GDNF (1:1000, SCBT), FGF-2 (1:500; Upstate), and IGF-1 (1:200: Millipore), as detailed in our previous publication [21, 22]. Confocal Z-sections (Olympus FV10i confocal microscope) were taken at one-micrometer intervals, and the percentages of graft-derived cells expressing GDNF, BDNF, FGF-2, or IGF1 were quantified. Approximately 200-400 graft-derived cells were analyzed from each grafted hippocampus (n=4-5 grafts) for every neurotrophic factor.

### Analyses of graft-mediated effects on host hippocampal interneurons

We examined whether NSC grafting intervention six days after SE would diminish the overall loss of host interneurons observed at a prolonged (9 months) period after SE in the host hippocampus. We processed two sets of serial sections (every 20^th^) through the hippocampus for neuropeptide Y (NPY) and parvalbumin (PV) immunostaining, using rabbit NPY (Peninsula labs) and mouse PV (Sigma Aldrich) antibodies, and quantified the numbers of NPY+ and PV+ interneurons using a stereological method. Interneurons expressing NPY or PV represent subclasses of GABA-ergic interneurons that display a substantial decline in number in the chronic phase after SE. The immunostaining and stereology methods employed for NPY and PV neuron counts are available in our previous report [13].

### Measurement of graft-mediated effects on normal and abnormal hippocampal neurogenesis

We quantified the status of host hippocampal neurogenesis in all groups through doublecortin (DCX) immunostaining of serial sections (every 15^th^; goat anti-DCX; 1:250; SCBT) and stereological quantification of DCX+ neurons in the subgranular zone-granule cell layer in the dentate gyrus. As per the timeline employed in this study, this measurement occurred at 9 months after SE in epileptic groups. Furthermore, to ascertain the effects of early NSC grafting on the quantity of abnormal neurogenesis (i.e., numbers of abnormally migrated granule cells born after SE) that occurs over 9 months' period after SE, we processed serial sections (every 20^th^) through the hippocampus for Prox-1 immunostaining using a rabbit Prox-1 antibody (1:1000; Millipore) and quantified the numbers of Prox1+ granule cells in the dentate hilus via stereology. As the extent of abnormal neurogenesis is linked to the loss of reelin+ interneurons, we processed another set of serial sections (every 20^th^) for reelin immunostaining using a rabbit reelin antibody (1:1000; Millipore) and quantified the numbers of reelin+ interneurons in the dentate hilus using stereology. The immunostaining and stereology methods employed for DCX, Prox-1, and Reelin are described in our previous reports [12, 21].

### Analysis of graft-mediated effect on aberrant mossy fiber sprouting in the dentate gyrus

Abnormal mossy fiber sprouting is characterized by the sprouting of dentate granule cell axons into the dentate supragranular layer (DSGL), one of the epileptogenic changes that progressively builds up after SE. To ascertain the effect of NSC grafting on this abnormal sprouting, we processed a set of serial sections (20^th^) for immunostaining using a rabbit ZnT3 antibody (1:250; Synaptic Systems) and quantified the area fraction occupied by the abnormal mossy fiber sprouting in the DSGL (n=4/group) using image J, as described in our previous publication [8, 40].

### Statistical Analyses

We employed one-way ANOVA with Newman-Keuls multiple comparison post hoc tests when three or more groups were compared, and two-tailed, unpaired, Student's t-test when two groups were compared. The numbers in bar charts are expressed as Mean ± S.E.M., and p<0.05 was considered significant.

## RESULTS

### NSC grafting after SE reduced SRS at 3 months post-SE and curbed the progression of SRS

**Figure 1.**
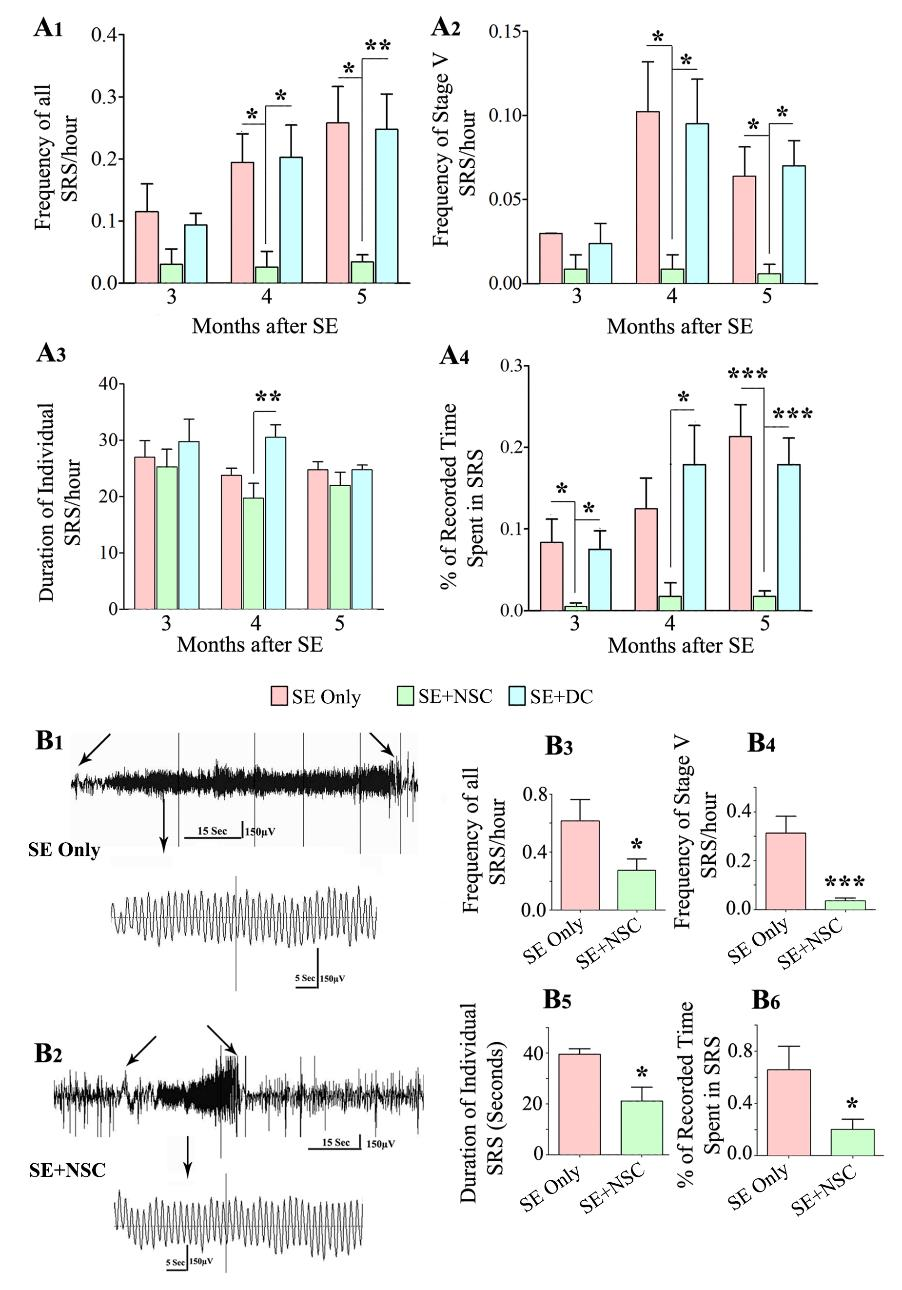
Early grafting of hippocampal neural stem cells (NSCs) reduced spontaneous recurrent seizures (SRS) at three months post-SE, curbed the progression of SRS at 3-5 months post-SE, and restrained the frequency and severity of SRS at 8 months post-SE as revealed by behavioral and EEG quantifications. The bar charts A1-A4 show the frequency of all SRS (A1), stage V SRS (A2), duration of individual SRS (A3), and the percentage of recorded time spent in SRS activity (A4). Note that the overall frequency and intensity of SRS as well as the percentage of time spent in SRS activity were significantly lower in the SE+NSC group (green), in comparison to the SE only(red) and SE+DC (blue) groups at 4 and 5 months post-SE (A1, A2, A4). All SRS activity parameters were mostly comparable between the SE+DC and SE only groups, implying that dead cell grafting after SE did not diminish or exacerbate SRS activity (A1, A2, A4). Also, note the progressive increase in SRS activity over 3-5 months in SE only and SE+DC groups compared to the SE+NSC group where SRS remained constant (A1-A4). Video-EEG recordings and analyses at 8 months post-SE revealed a sustained reduction in SRS at an extended time point after SE (B1-B6). EEG tracings in B1 and B2 show the reduced severity of SRS activity in the SE+NSC group (B2) in comparison to the SE alone group (B1). The bar charts (B3-B6) compare the various EEG data between the SE only and SE+NSC groups at 8 months after SE, which revealed significantly reduced SRS activity for all measured parameters. * p < 0.05. **p < 0.01, *** p < 0.001.

We analyzed the frequency and intensity of behavioral SRS, the duration of individual SRS, and the percentage of time spent in SRS activity at 3, 4, and 5 months after SE in all three SE groups: SE only, SE+NSC and SE+DC groups (n = 6-8/group, Fig. 1A1-A4). The overall frequency and intensity of SRS, as well as the percentage of time spent in SRS activity, were lower in the SE+NSC group, in comparison to SE only and SE+DC groups (Fig. 1A1, A2, A4). First, one-way ANOVA analysis was performed for each parameter of SRS data every month. The differences in all SRS and stage-V SRS were significant at 4- and 5-months post-SE (p<0.01-0.05, Fig. 1A1-A2). In comparison to SE only and SE+DC groups, the SE+NSC group displayed an 87% reduction in all SRS, and 91-92% reduction in stage-V SRS at 4 months post-SE, and 91-92% reductions in all SRS and stage-V SRS at 5 months post-SE. (p<0.01-0.05, Fig. 1A1-A2). The duration of individual seizures was mostly comparable between groups except at 4 months post SE, where animals in the SE+NSC group displayed a 35% reduction in the duration of individual seizures in comparison to animals in the SE+DC group (p<0.05; Fig. 1A3). However, the differences in the overall time spent in seizure activity were significant between groups at 3, 4, and 5 months after SE (p<0.001-0.05, Fig. 1A4). In comparison to SE only and SE+DC groups, the SE + NSC group displayed a 94% reduction at 3 months, 86-91% reduction at 4 months, and 92% reduction at 5 months (p<0.001-0.05, Fig. 1A4). Next, repeated-measures ANOVA was performed on each SRS parameter to see the progression of SRS over 3-5 months after SE. In the SE alone group, the frequencies of all SRS and the percentage of times spent in SRS activity progressively increased over 3-5 months (p<0.01-0.05, Fig. 1 A1, A4). The SE+DC group also showed a similar trend, but increases were not significant statistically (p>0.05, Fig. 1 A1, A4). In contrast, frequencies of all SRS, stage-V SRS, and the amount of time spent in SRS activity remained constant over 3-5 months in the SE+NSC group (p>0.5). Thus, grafting of hippocampal NSCs early after SE reduced the occurrence of SRS at 3 months post-SE and restrained the progression of SRS at 3-5 months post-SE. Also, all SRS activity parameters were comparable between SE+DC and SE only groups, implying that dead cell grafting after SE did not diminish or exacerbate SRS activity.

### NSC grafting-mediated reduced SRS persists for prolonged periods after SE

We determined whether the reduced SRS seen after NSC grafting at 3-5 months post-SE is sustained at an extended time point after SE. We measured different parameters of SRS at 8 months after SE in the SE only group (n=4) and the SE+NSC group (n=8), using continuous video-EEG recordings (Fig. 1B1-B6). Such video-EEG analysis demonstrated significantly reduced SRS activity for all measured parameters in the SE+NSC group, compared to the SE only group. The seizures were also reduced in terms of severity in the SE+NSC group, which can be seen from the comparison of representative EEG traces during SRS between an animal from the SE only group (Fig. 1B1) and an animal from the SE+NSC group (Fig. 1B2). The overall reductions in the SE+NSC group were 57% for the frequency of all SRS, 89% for stage-V SRS, 46% for the duration of individual SRS, and 70% for the percentage of times spent in SRS activity (p<0.001-0.05, Fig. 1B3-B6). Thus, grafting of hippocampal NSCs early after SE leads to reduced SRS activity even at an extended time-point of 8 months after SE, implying that NSC grafting -mediated reduced SRS endures for prolonged periods after SE.

**Figure 2.**
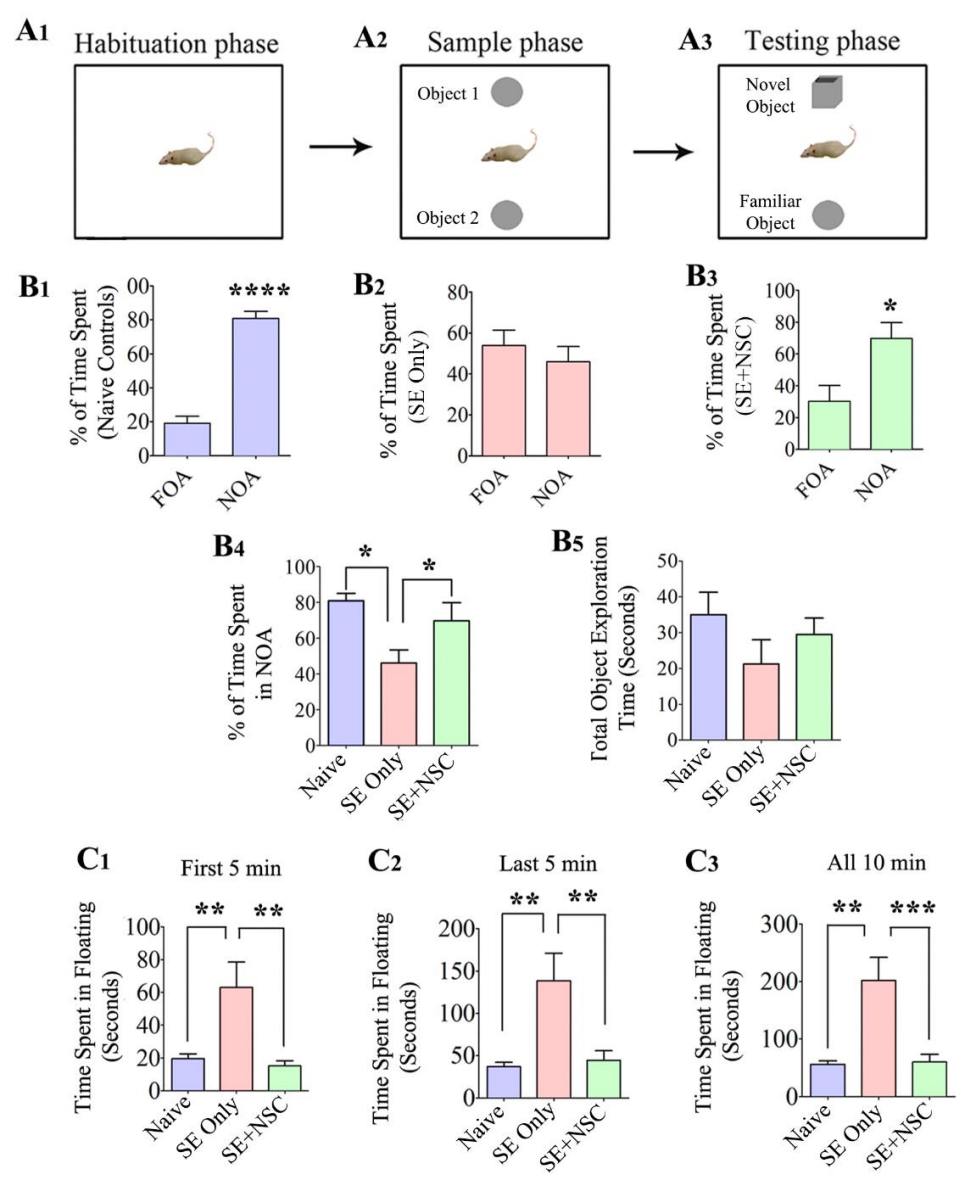
Early intervention with hippocampal NSC grafting after SE preserved recognition memory function and diminished the depressive-like behavior during the chronic phase of epilepsy. A Novel Object Recognition test (NORT) was used for this test. The cartoon A1-A3 shows the open field box with different objects during the three phases of this test. The bar charts B1-B3 demonstrate the performance of animals in the naïve control (purple), SE only (red), and SE+NSC group (green). Note a significantly higher preference of animals in the naïve control and SE+NSC groups to explore novel object area (NOA) over the familiar object area (FOA) in trial 3 (B1, B3, B4), which implied an ability for recognition memory. In contrast, the animals in the SE only group did not show any preference for either the FOA or NOA in Trial 3 (B2, B4). Note that the total object exploration times were comparable between the three groups (B5). The bar charts C1-C3 show the extent of depressive-like behavior in different groups in a forced swim test (FST). The total time spent in immobility during the FST was used as a measure of depressive-like behavior. Note that the times spent in floating were significantly greater in SE only animals at first 5 minutes (C1), last 5 minutes (C2), or for the entire duration of 10 minutes (C3). In contrast, the duration of immobility in the SE+NSC group was highly comparable to that of animals in the naïve control group but significantly lower than the SE only group in different segments and during the entire duration of the test, indicating a graft-mediated reduction in depressive-like behavior (C1-C3). *p<0.05; **p<0.01; ***p<0.001.

### NSC grafting after SE preserved recognition memory function in the chronic epilepsy phase

Animals belonging to different groups (n=6/group) were interrogated for recognition memory function at ~6 months post-SE with a novel object recognition test (NORT), which examined the ability of animals to distinguish a novel object from a familiar object [21, 36]. The test comprised three successive trials of 5 minutes duration each (T1-T3), in which animals explored an empty open field apparatus (T1-Habitation phase), two identical objects placed on opposite sides of the open field (T2-Sample phase) and one familiar object (i.e., one of the objects from T2 remaining in its location) and a novel object (T3-Testing phase). (Fig. 2A1-A3). The ratio of the total object exploration time spent with the novel object in the testing phase of NORT was used as a recognition memory measure. The naïve control animals showed a maximum preference for exploring the novel object area (NOA) over the familiar object area (FOA) in T3 (p<0.0001, Fig. 2B1), implying a robust recognition memory. The animals in the SE only group did not show any preference for either the FOA or NOA in T3 (p>0.05, Fig. 2B2), suggesting recognition memory dysfunction in these animals. In contrast, the behavior of animals in the SE+NSC group was closer to naïve control animals, which was evident from their preference to explore the NOA over the FOA in T3 (p<0.05, Fig. 2B3). A separate comparison of the percentages of time spent in the NOA across groups also demonstrated the beneficial effect of NSC grafting in preserving the recognition memory function after SE (Fig. 2B4). The total object exploration times did not differ significantly between the three groups, however (p>0.05, Fig. 2B5). Thus, NSC grafting early after SE leads to the maintenance of better memory function in the chronic epilepsy phase.

### NSC grafting after SE prevented depressive-like behavior in the chronic epilepsy phase

We also evaluated the extent of depressive-like behavior in animals belonging to the SE only and SE+NSC groups (n=7-8/group) at ~6 months post-SE through an FST. The total time spent in immobility or floating in FST was used as a measure of depressive-like behavior. The animals in the SE only group spent ~4 times higher amount of time in floating than naïve control animals, which implied depressive-like behavior. The times spent in floating was significantly higher in SE only animals when data were analyzed for different segments (first or last 5 minutes) or for the entire duration of 10 minutes (p<0.01, Fig 2C1-C3). In contrast, the times spent in floating in the SE+NSC group were highly comparable to animals in the naïve control group, but significantly lower than the SE only group in different segments as well as when the entire duration of the test was analyzed in entirety (p<0.01, Fig. 2C1-C3). Thus, NSC grafting after SE prevents the development of depressive-like behavior in the chronic phase of epilepsy.

### Cells from NSC grafts displayed long-term survival in the SE-injured hippocampus

**Figure 3.**
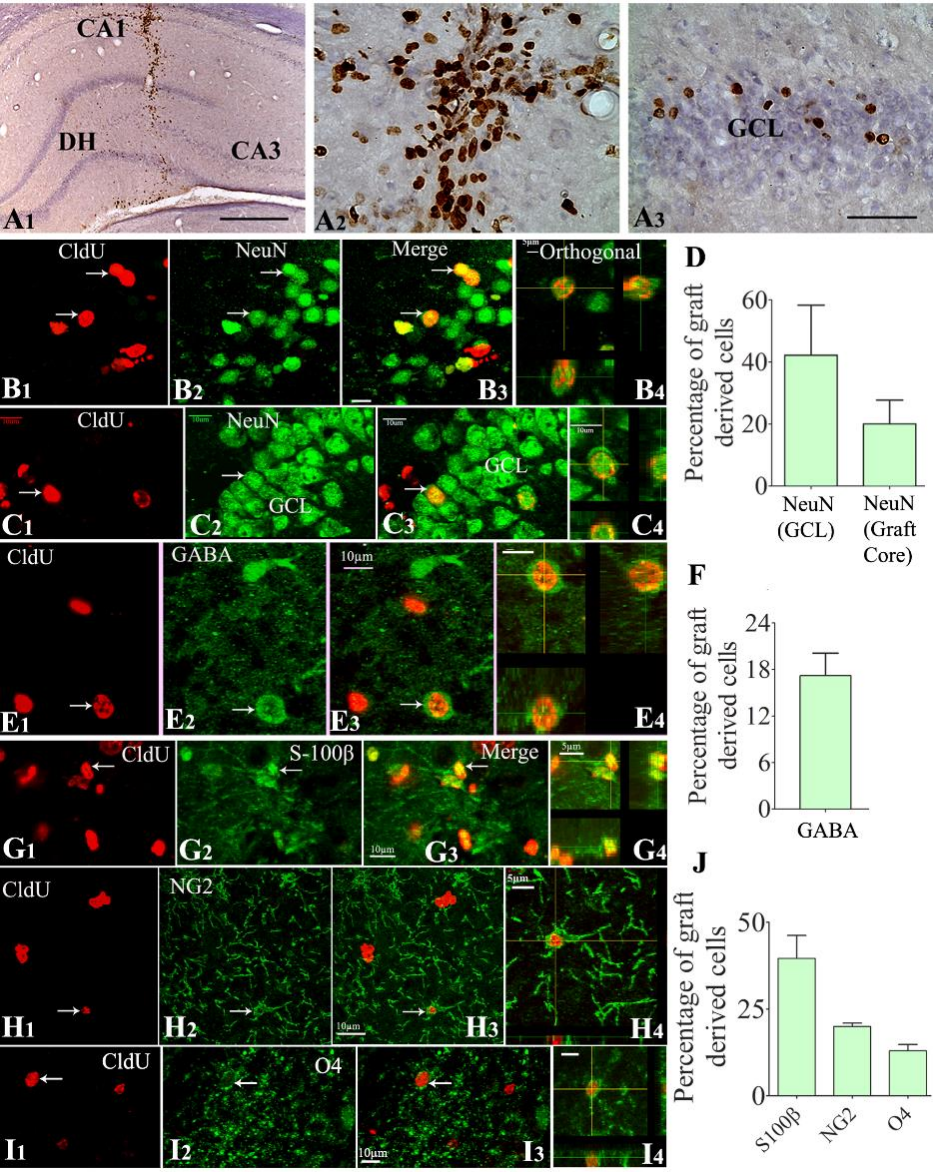
Cells derived from neural stem cell (NSC) grafts displayed long-term survival and differentiated into all three neural cell types in the host hippocampus that underwent SE-induced injury. The top panel (A1-A3) shows the 5'-Chloro-2'-deoxyuridine CldU-positive graft-derived cells in the host hippocampus at ~9 months post-grafting (A1). A2 and A3 are magnified views of regions from A1, depicting the distribution of CldU+ cells in the graft core (A2) and neighboring dentate granule cell layer (GCL, A3). B1-C4 demonstrate samples of confocal Z section images visualizing graft-derived neurons positive for CldU and NeuN in the CA3 region (B1-B3) and the GCL (C1-C3). B4 and C4 show orthogonal views of graft-derived neurons expressing CldU-NeuN. The bar chart D depicts the overall neuronal differentiation of graft-derived cells in the GCL (neurogenic) and the graft core (D). Figures E1-E4 demonstrate graft-derived CldU+ cells expressing gamma-aminobutyric acid (GABA). E4 shows an orthogonal view of a graft-derived interneuron expressing CldU and GABA. The bar chart F depicts the overall % of graft-derived cells differentiating into GABA-ergic interneurons. Figures G1-I4 show the differentiation of graft-derived CldU+ cells into S-100ß+ mature astrocytes (G1-G3), NG2+ oligodendrocyte progenitors (G1-G3), and O4+ oligodendrocytes (I1-I3) in the host hippocampus. G4, H4, and I4 show the orthogonal view of a graft-derived astrocyte (G4), an oligodendrocyte progenitor cell (H4), and an oligodendrocyte (I4). The bar chart J shows the percentage of S-100ß+ astrocytes, NG2+ oligodendrocyte progenitors, and oligodendrocytes among graft-derived cells. Scale bars: A1, 200 µm; A2 and A3, 50 µm; B1-E4, G1-G3, H1-H3, and I1-I4, 10 µm; G4 and H4, 5 µm. DH, dentate hilus.

The cells derived from NSC grafts placed into the hippocampus after SE were visualized by CldU immunohistochemistry at ~9 months after grafting. The majority of graft-derived cells (i.e., CldU+ cells) were found in the graft core, a region containing densely packed graft-derived cells located at the site of graft injection. However, smaller fractions of graft-derived cells migrated to neighboring cell layers of the hippocampus, which include the CA1 cell layer, the granule cell layer, the dentate hilus (DH), and the CA3 cell layer (Fig. 3A1-A3). Stereological quantification of graft-derived CldU+ cells in serial sections through the entire hippocampus revealed a yield equivalent to ~35% (34.6±4.0%) of injected cells (n=5). Since four grafts (~80,000 live cells/graft) were placed into each hippocampus (i.e., ~320,000 live cells/hippocampus), the 35% graft cell yield meant 9 months survival of ~112,000 graft-derived cells in each hippocampus that underwent SE-induced injury. The actual addition of graft-derived cells into the host hippocampus is likely higher than the estimated number because it is plausible that some of the graft-derived cells have lost their CldU label due to the proliferation of NSCs more than a few times after grafting.

### Cells from NSC grafts differentiated into NeuN+ mature neurons and GABA+ interneurons

Z-section analysis of brain tissue sections processed for CldU and NeuN dual immunofluorescence in a confocal microscope demonstrated the presence of mature neurons among graft-derived cells. Such graft-derived mature neurons were found in the graft cores as well as among graft-derived cells that migrated into the CA3 pyramidal cell layer (Fig. 3B1-B4) or the dentate granule cell layer (Fig. 3C1-C4]. The overall differentiation of graft-derived cells into neurons was ~20% in graft cores and among migrated graft-derived cells in the CA1 or CA3 region (n=8). Interestingly, ~42% of graft-derived cells that migrated into the granule cell layer differentiated into NeuN+ neurons (Fig. 3D). Z-section analysis of brain tissue sections processed for CldU and GABA dual immunefluorescence revealed the occurrence of GABA-ergic interneurons among graft-derived cells that migrated into different hippocampal regions (Fig. 3E1-E4). The overall differentiation of graft-derived cells into GABA-ergic interneurons was ~17% (Fig. 3F, n=7). Extrapolation of the yield of graft-derived cells in each hippocampus with percentages of NeuN+ and GABA+ neurons among graft-derived cells suggested that NSC grafting resulted in the addition of ~21,280 NeuN+ neurons and ~19,040 GABA+ interneurons to each hippocampus.

### Cells from NSC grafts differentiated into astrocytes, oligodendrocyte progenitors, and oligodendrocytes

Measurements from brain tissue sections processed for CldU-S100β, CldU-NG2, or CldU-O4 dual immunofluorescence and Z-section analyses revealed the presence of S100β+ mature astrocytes (Fig. 3G1-G4), NG2+ oligodendrocyte progenitors (Fig. 3H1-H4), and O4+ oligodendrocytes (Fig. 3I1-I4) among graft-derived cells. Quantification revealed that, among graft-derived cells, ~40% differentiated into S-100β+ astrocytes, 20% into NG2+ oligodendrocyte progenitors, and ~13% into O4+ oligodendrocytes (Fig. 3J, n=4-8). Extrapolation of the yield of graft-derived cells in each hippocampus with percentages of S100β+, NG2+, and O4+ cells suggested that NSC grafting resulted in the addition of ~44,800 mature astrocytes, ~22,400 oligodendrocyte progenitors, and ~14,560 mature oligodendrocytes to each hippocampus.

### Cells derived from NSC Grafts expressed diverse neurotrophic factors

**Figure 4.**
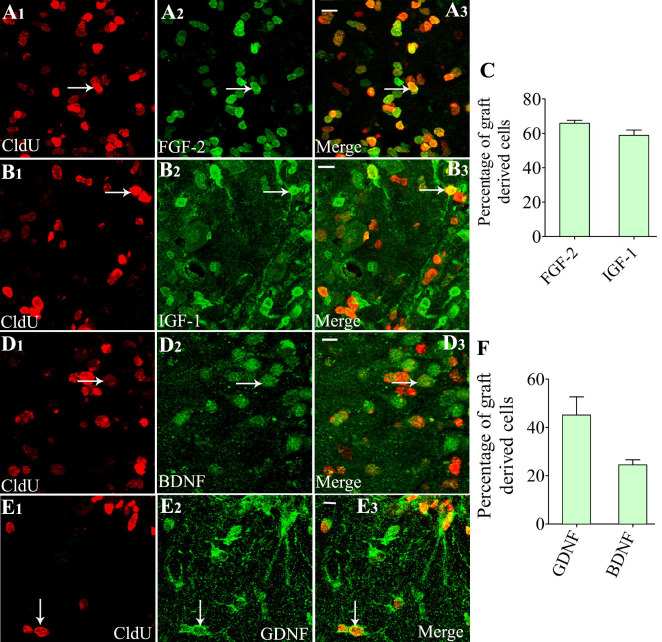
Cells derived from neural stem cell (NSC) grafts expressed multiple neurotrophic factors in the host hippocampus when examined at 9 months post grafting. A1-E3 show dual immunofluorescence confocal images for 5'-Chloro-2'-deoxyuridine (CldU) and fibroblast growth factor-2 (FGF-2) (A1-A3), CldU and insulin-like growth factor-1 (IGF-1) (B1-B3), CldU and brain-derived neurotrophic factor (BDNF) (D1-D3), and CldU and glial cell line-derived neurotrophic factor (GDNF) (E1-E3). The bar chart C depicts the % of the graft-derived cells expressing FGF-2 and IGF-1, whereas the bar chart F shows the percentages of graft-derived cells expressing BDNF and GDNF. White arrows in A1-E3 show examples of dual-labeled cells. Scale bars: = A3, B3, D3, and E3, 10 µm.

Z-section analysis of brain tissue sections processed for CldU-FGF-2, CldU- IGF-1, CldU-BDNF, or CldU- GDNF dual immunofluorescence demonstrated that variable fractions of graft-derived cells displayed the expression of FGF-2 (Fig. 4A1-A3), IGF-1 (Fig. 4B1-B3), BDNF (Fig. 4 [D1-D3]), or GDNF (Fig. 4E1-E3). Quantification revealed that higher percentages of graft-derived cells expressed FGF-2 (~66%) and IGF-1 (~59%) (Fig. 4C), and relatively lower percentages of graft-derived cells expressed BDNF (~25%) and GDNF (~45%) (Fig. 4F, n=4).

### NSC grafting after SE preserved higher numbers of interneurons

**Figure 5.**
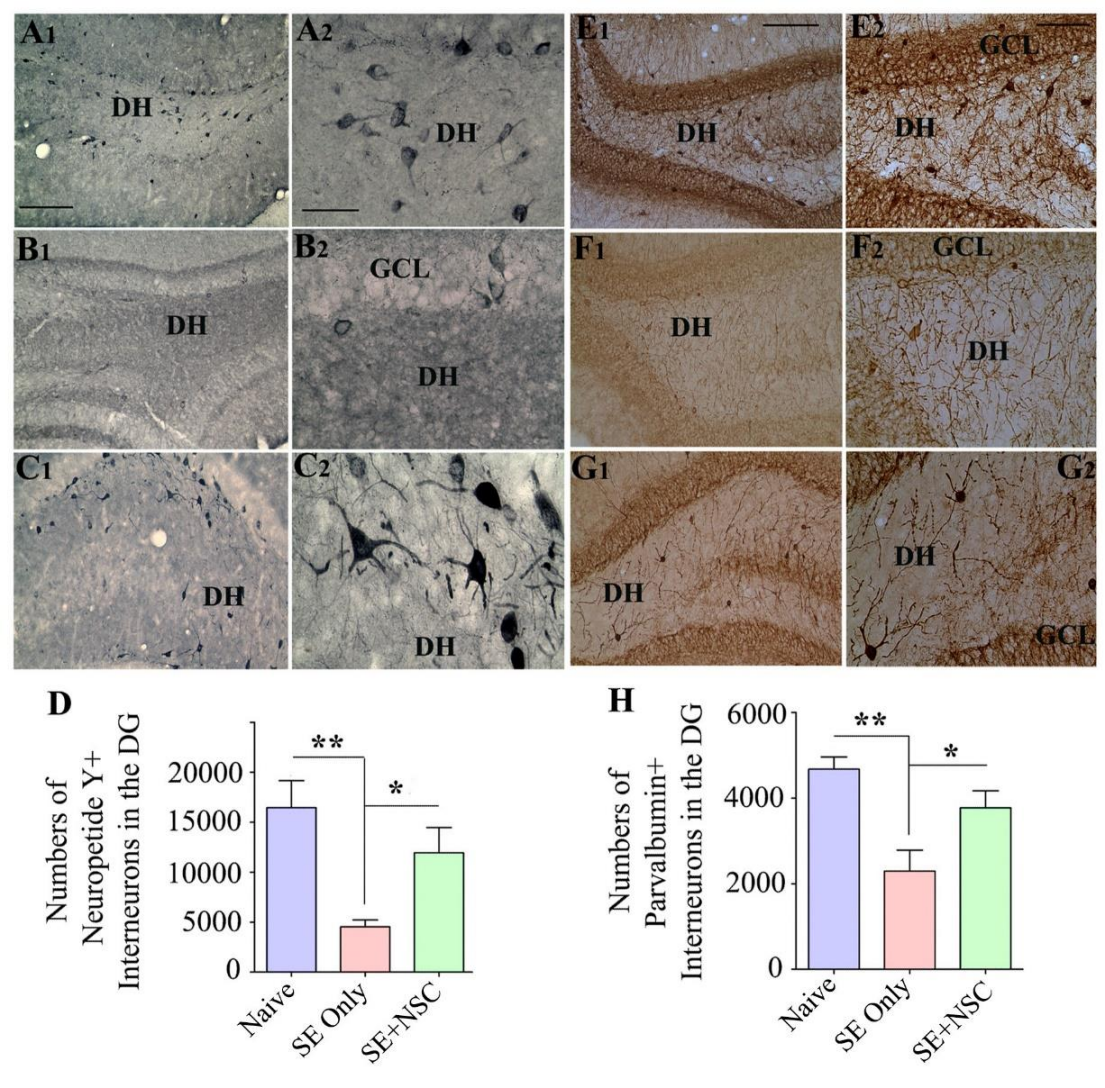
Early neural stem cell (NSC) grafting after SE preserved higher numbers of interneurons in the dentate hilus (DH) of the host hippocampus. The panels on the left show the neuropeptide Y (NPY)-positive interneurons in the dentate gyrus (DG) of animals belonging to the naïve control (A1), SE only (B1), or SE+NSC (C1) groups. A2, B2, and C2 are magnified views of regions from A1, B1, and C1, respectively, showing the morphology of NPY+ interneurons. Note significant preservation of NPY+ neurons in the SE+NSC group exhibiting hypertrophy (C2). The bar chart D compares the number of NPY+ interneurons in the DG between different groups. The panels on the right show the parvalbumin (PV)-positive interneurons in the DG of animals belonging to naïve control (E1), SE only group (F1), or SE+NSC (G1) groups. E2, F2, and G2 are magnified views of regions from F1, G1, and H1, respectively. The Bar chart H compares the number of PV+ interneurons in the DG between different groups. *, p<0.05; **, p<0.01. Scale bars: A1, B1, C1, E1, F1, and G1, 200 µm; A2, B2, and C2, 50 µm; E2, F2, and G2, 100 µm. GCL, granule cell layer.

We examined the effects of early NSC grafting after SE on the preservation of interneurons in the DH at ~9 months post-SE through immunohistochemistry for NPY and PV. In comparison to naïve control animals, the SE only group displayed a higher loss of NPY+ interneurons than the SE+NSC group (Fig. 5A1-C2). Stereological quantification using serial sections through the entire hippocampus demonstrated that, in comparison to the naïve control group, there was a 72% decline in NPY+ interneurons in the SE only group (p<0.01) in contrast to only a 27% reduction in the SE+NSC group (p>0.05, Fig. 5D). Moreover, the NPY+ interneuron population in the SE+NSC group was 3.6 folds higher than the SE only group (p<0.05, Fig. 5D, n=5/group). The soma of many NPY+ neurons in the SE+NSC group also exhibited hypertrophy (Fig. 5C2), likely implying enhanced activity of these interneurons in the chronic phase of epilepsy. Immunostaining for PV+ interneurons also showed a similar trend as NPY+ interneurons (Fig. 5E1-G2). The stereological measurement revealed that, in comparison to the naïve control group, there was a 49% decline in PV+ interneurons in the SE only group (p<0.01) vis-à-vis 19% reduction in the SE+NSC group (p>0.05, Fig. 5 [H], n=5/group). Also, the PV+ interneuron population in the SE+NSC group was 1.6 folds higher than the SE only group (p<0.05, Fig. 5H). Thus, NSC grafting after SE preserved higher numbers of NPY+ and PV+ interneurons in the DH.

**Figure 6.**
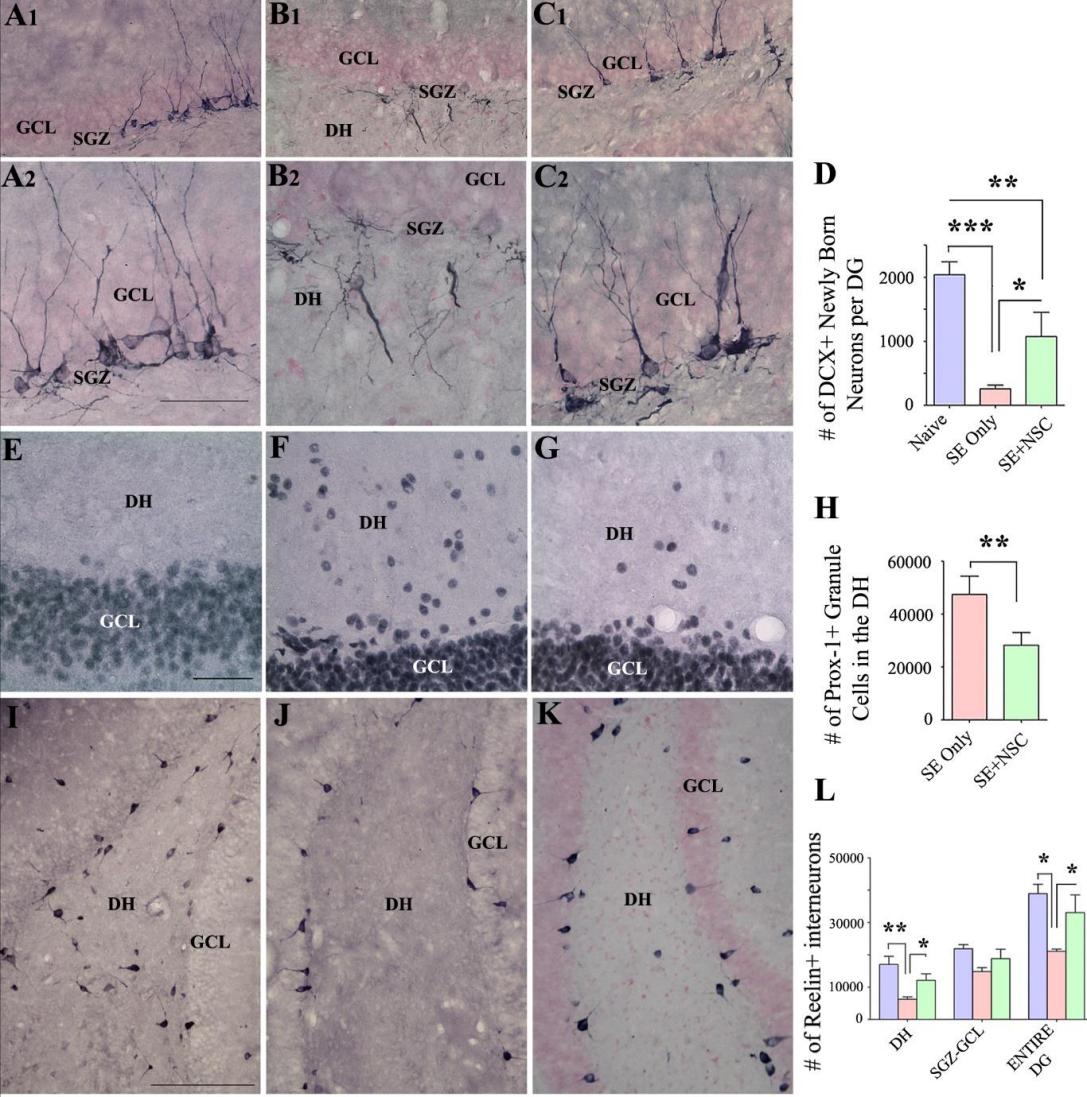
Neural stem cell (NSC) grafting after SE promoted a higher level of normal neurogenesis and reduced the aberrant neurogenesis in the DG during the chronic epilepsy phase. The top panel shows the doublecortin (DCX)-positive newly born neurons in the DG of animals belonging to the control (A1), SE only (B1), and SE+NSC (C1) groups. A2, B2, C2 are magnified views of regions from A1, B1, C1, respectively. Note the dramatically declined normal neurogenesis and prominent aberrant neurogenesis in the dentate hilus (DH) of SE only group(B1-B2) and a preserved dentate neurogenesis and reduced abnormal neurogenesis in the SE+NSC graft group (C1-C2). The bar chart D compares the number of DCX+ neurons in the dentate gyrus (DG) between the three groups. Note a substantially declined normal dentate neurogenesis in the SE only group, in comparison to the naïve group and a much higher level of neurogenesis in the SE+NSC group (D) at ~9 months post-SE. The panel E-G shows prox-1+ dentate granule cell in the DH of animals belonging to the naïve control (E), SE only (F), and SE+NSC (G) groups. The bar chart H compares the number of prox1+ cells in the DH between the three groups. Note significantly reduced Prox1+ cells in the SE+NSC group, implying the long-term benefits of grafting in reducing the extent of abnormal dentate neurogenesis. The lower panel (I, J, K) shows reelin+ interneurons in animals belonging to the naïve control (I), SE only (j), and SE+NSC (K) groups. Bar chart L compares reelin+ interneurons between the three groups. Note that, in comparison to SE only group, the SE+NSC group displayed better preservation of reelin+ interneurons in the DH and when the entire DG is taken in its entirety (L). *p<0.05; **p<0.01; ***p<0.001. Scale bars, I, J, and K, 200 µm, A1, B1, and C1, 100 µm, A2, B2, C2, E, F, and G, 50 µm. GCL, granule cell layer; SGZ, Subgranular zone.

### NSC grafting after SE promoted normal neurogenesis and reduced aberrant neurogenesis

The response of dentate neurogenesis to an incidence of SE is typically biphasic, which is characterized by increased neurogenesis with a significant amount of aberrant neurogenesis in the acute and subacute phases [11, 42, 43], and much-waned neurogenesis in the chronic phase [43, 44]. We investigated whether early NSC grafting after SE would preserve a significant amount of normal neurogenesis for prolonged periods as well as reduce the aberrant neurogenesis at ~9 months post-SE, using doublecortin (DCX) immunostaining of brain tissue sections. In comparison to the naïve control group, the SE only group displayed substantially declined neurogenesis than the SE+NSC group (Fig. 6 A1-C2). Also, the extent of aberrant neurogenesis, typified by the migration of newly born neurons into the DH, was highly prominent in the SE only group but negligible in the SE+NSC group (Fig. 6B1-B2, C1-C2). Stereological quantification revealed that, in comparison to the naïve control group, there was an 87% decline in DCX+ newly born neurons in the SE only group (p<0.001) in contrast to 47% reduction in the SE+NSC group (p<0.01, Fig. 6D, n=6/group). Moreover, the DCX+ newly born neurons in the SE+NSC group was 4.2 folds higher than the SE only group (p<0.05, Fig. 6D). Thus, the SE+NSC group maintained a much higher level of neurogenesis than the SE only group at ~9 months post-SE. Also, much of the neurogenesis in the SE+NSC group was normal, in comparison to mostly abnormal neurogenesis in the SE only group.

**Figure 7.**
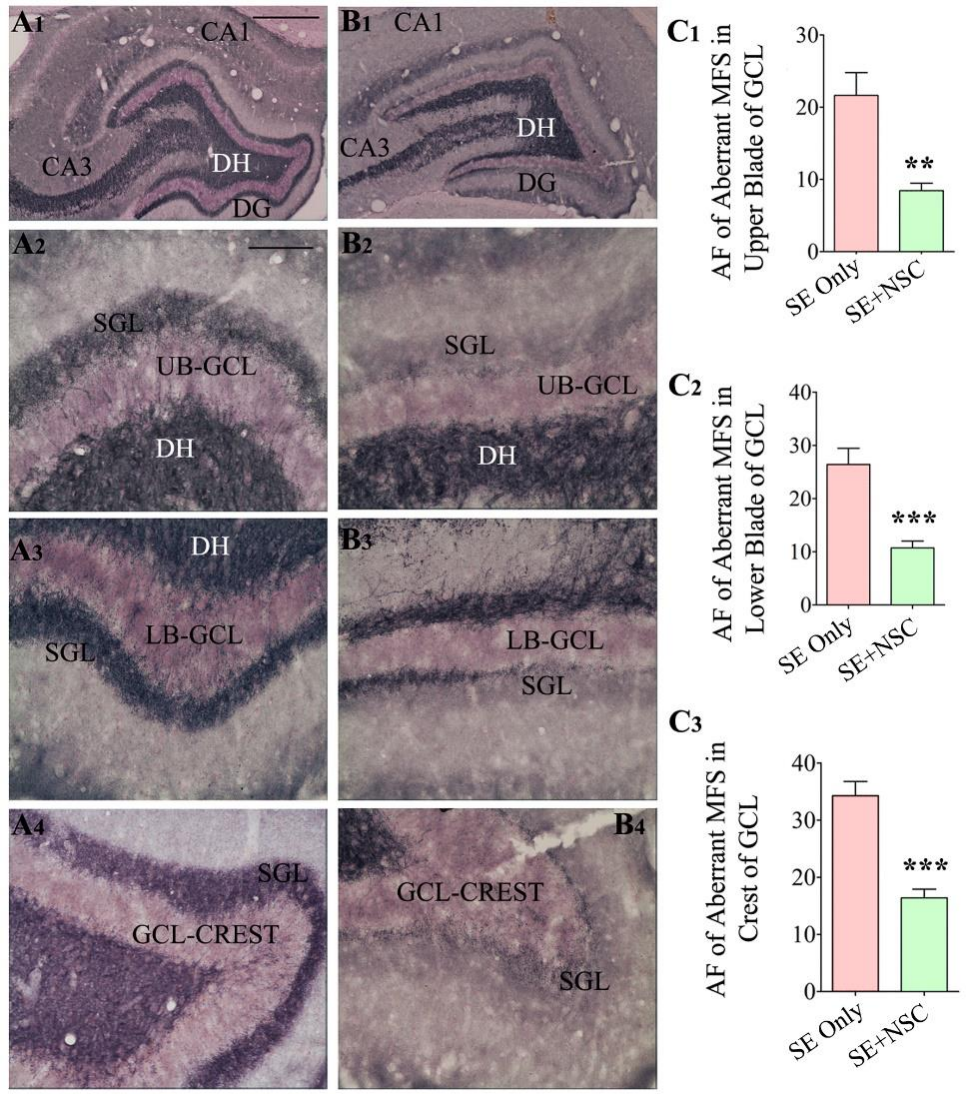
Neural stem cell grafting after SE reduced the extent of aberrant mossy fiber sprouting into the dentate supragranular layer (DSGL) when examined at 9 months post-SE. The ZNT-3 immunostaining was performed to visualize the extent of aberrant mossy fiber sprouting in the SE only (A1-A4) and SE+NSC groups (B1-B4). Note the highly conspicuous aberrant mossy fiber sprouting with dark bands in the upper blade (A2), the lower blade (A3), and the crest (A4) of the granule cell layer (GCL) in the SE only group, and greatly diminished sprouting in the SE+NSC group (B2, B3, B4). The bar charts C1, C2, C3 compare the area fraction of sprouted mossy fibers between the SE only and SE+NSC groups. Note that the intervention with hippocampal NSC grafting has significantly reduced the extent of aberrant mossy fiber sprouting in all three regions of the dentate gyrus (DG) in comparison to the SE only group (C1-C3). **, p<0.01; ***, p<0.001. Scale bars: A1 and B1, 500 µm, A2-B4, 100 µm. AF, area fraction; DH, dentate hilus; GCL, granule cell layer; SGL, supragranular layer; UB, upper blade; LB, lower blade.

We also investigated the occurrence of granule cells in the DH using immunostaining for prox-1 to gauze the extent of abnormal neurogenesis during the nine months after SE. The occurrence of prox-1+ granule cells in the DH was rare in the naïve control group, highly conspicuous in the SE only group, and much reduced in the SE+NSC group (Fig. 6E-G). Stereological quantification revealed that the SE only group displayed 1.7 folds the higher number of prox-1+ granule cells in the DH than the SE+NSC group (p<0.01, Fig. 6H), n=7/group), implying that NSC grafting reduced the extent of abnormal neurogenesis that occurred during the nine months after SE. Consistent with the prox-1 results, the SE+NSC group displayed better preservation of reelin+ interneurons in the DG than the SE only group (Fig. 6I-L). Stereological quantification revealed that, in comparison to the naïve control group, there was a 46% decline in reelin+ interneurons in the SE only group (p<0.05) in contrast to only a 15% reduction in the SE+NSC group (p>0.05, Fig. 6L). Moreover, the reelin+ interneurons in the SE+NSC group were 1.6 folds higher than the SE only group (p<0.05, Fig. 6L). Since the loss of reelin-secreting interneurons in the DG is implicated in the abnormal resettlement of newly born neurons into the DH after SE [45], the preservation of higher numbers of reelin+ interneurons likely underlie the reduced number of prox1+ granule cells in the DH of the SE+NSC group.

**Figure 8.**
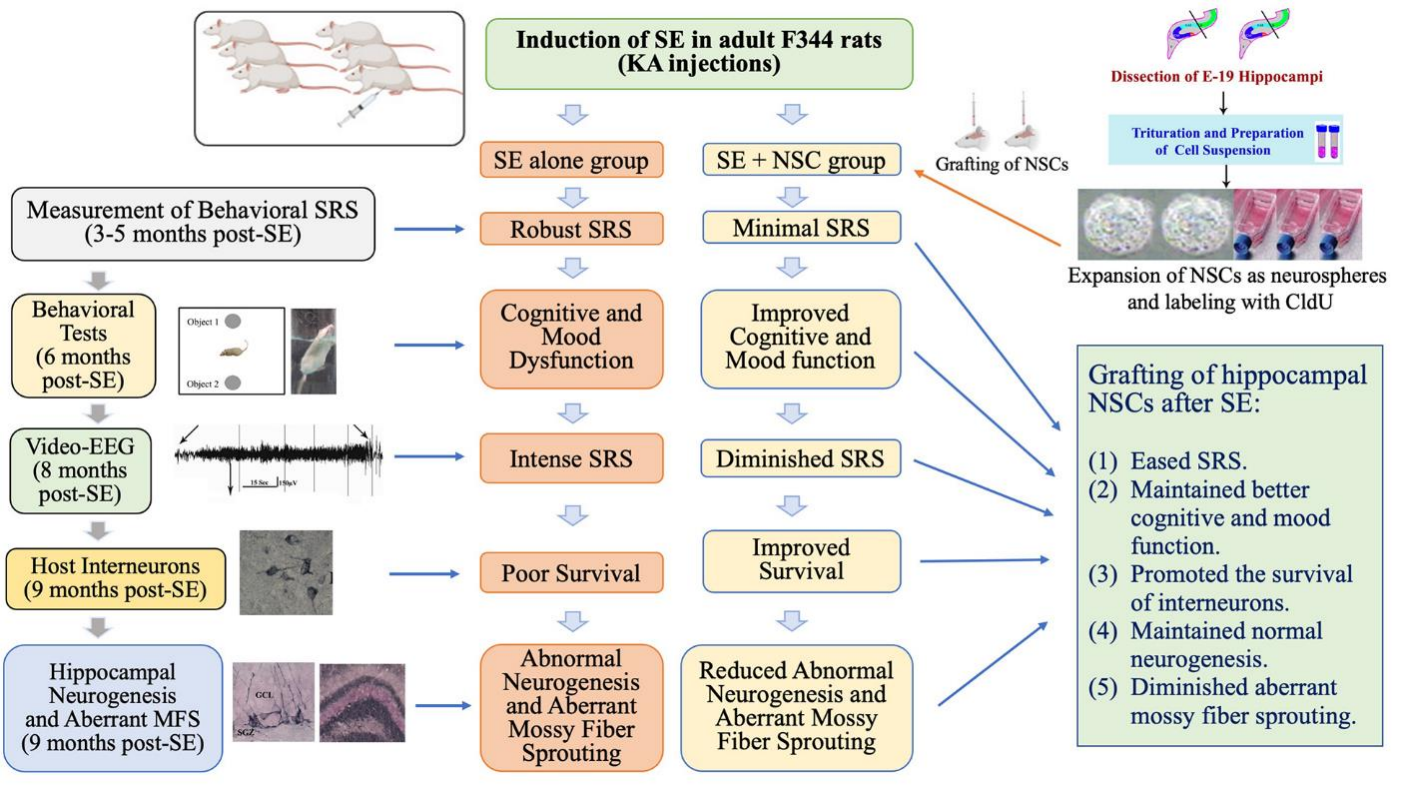
The cartoon depicts a summary of the experimental design, results, and significant findings. The top portion shows the induction of status epilepticus (SE) in F344 rats through kainic acid (KA) injections. The top right side of the figure illustrates the dissection of embryonic day 19 (E19) rat hippocampi, trituration of hippocampal tissues, expansion of neural stem cells (NSCs) as neurospheres, and labeling of NSCs with 5'-Chloro-2'-deoxyuridine (CldU) in vitro. Neurospheres were triturated into a cell suspension before grafting into the hippocampus at six days post-SE. The left side of the figure shows the timeline for various analyses performed in the study, whereas the middle portion of the figure illustrates multiple changes in the SE alone group and the SE + NSC group. The summary of results is listed on the lower right portion of the figure.

### NSC grafting after SE reduced the extent of aberrant mossy fiber sprouting

We examined the extent of aberrant mossy fiber sprouting into the dentate supragranular layer (DSGL) of animals in both SE only and SE+NSC groups through ZNT-3 immunostaining. The aberrant mossy fiber sprouting appeared highly conspicuous with dark bands in the upper and lower blades as well as the crest of the dentate granule cell layer in the SE only group (Fig. 7A1-A4) but seemed to be diminished in the SE+NSC group (Fig. 7B1-B4). Quantification of the area fraction of mossy fibers in the DSGL revealed that compared to the SE only group, the extent of aberrant sprouting was significantly reduced in the SE+NSC group in all regions of the dentate granule cell layer. The overall reductions were 61% in the upper blade (p<0.01, Fig. 7C1), 59% in the lower blade (p<0.001, Fig. 7C2), and 52% in the crest (p<0.001, Fig. 7C1, n=5-6/group). Thus, early NSC grafting after SE reduced the occurrence of aberrant mossy fiber sprouting into the DSGL.

## DISCUSSION

The present study demonstrated that bilateral grafting of hippocampal NSCs into hippocampi after SE-induced injury is efficacious for alleviating the frequency and severity of SRS and preserving cognitive and mood function for prolonged periods after SE. Remarkably, such therapeutic effects were associated with modulation of the abnormal plasticity and interneuron loss in the DG. These include higher levels of normal neurogenesis, diminished abnormal migration of newly born neurons, repression of aberrant mossy fiber sprouting, and better preservation of NPY-, PV- and reelin-positive interneurons. Moreover, grafts survived for nine months in the hippocampus with differentiation of graft-derived cells into neurons, astrocytes, oligodendrocytes, and a significant number of GABA-ergic interneurons. Significant fractions of graft-derived cells also expressed beneficial neurotrophic factors. These findings are also summarized in the graphical abstract (Fig. 8).

### Potential mechanisms of reduced SRS activity after NSC grafting

At 4-5 months after SE, in comparison to control groups, animals receiving hippocampal NSC grafts displayed significantly reduced behavioral SRS activity. Interestingly, at 3-5 months post-SE, the frequency of SRS progressively increased in SE alone animals but not in SE animals receiving NSC grafts, implying that NSC grafting after SE curbed the advancement of epileptogenic processes. Moreover, continuous video-EEG recordings performed at 8 months after SE demonstrated that animals receiving NSC grafts continued to display reduced seizure activity compared to SE only animals. These findings imply that grafting of hippocampal NSCs early after SE slows down epileptogenic processes, eventually culminating in a much reduced and less severe chronic epileptic state at 8 months post-SE.

Enduring reductions in the frequency and intensity of SRS in animals receiving hippocampal NSC grafts likely involved multiple mechanisms. A comparison of various alterations between the untreated epileptic hippocampus and the epileptic hippocampus that received NSC grafts provided insights on potential mechanisms. NSC grafting resulted in the addition of new cells and proteins to the epileptic hippocampus, which could mediate anti-seizure activity. The addition of ~19,000 new GABA-ergic interneurons to each epileptic hippocampus has likely contributed to reduced SRS activity because GABA-ergic interneuron loss is one of the factors contributing to the occurrence of SRS in chronic TLE [20, 34, 41, 46-49]. Also, intrahippocampal grafting of interneuron precursors has consistently resulted in diminished SRS activity in models of SE or chronic epilepsy [11, 34, 50-52]. Significant fractions of transplant-derived cells expressed several neurotrophic factors having anticonvulsant properties such as GDNF, IGF-1, and FGF-2, which might have resulted in higher concentrations of anti-seizure proteins in the milieu of the epileptic hippocampus. Indeed, the role of these graft-derived neurotrophic factors in seizure suppression is corroborated by findings that seizure activity declined when the epileptic hippocampus was genetically or pharmacologically manipulated to contain higher concentrations of GDNF [53-55], IGF-1 [56] or FGF-2 [57]. Thus, NSC grafting resulted in an improved anti-seizure defense in the epileptic hippocampus.

NSC grafting also positively modulated the abnormal plasticity that typically ensues after SE in the hippocampus. In the absence of treatment, neurogenesis in the DG becomes abnormal after SE, which is typified initially by the overproduction and abnormal migration and integration of newly born neurons, and later by much-waned neurogenesis [10, 42-44]. Substantially declined neurogenesis could increase SRS activity in the chronic phase because studies have shown that the intensity of SE varies depending on the extent of normal neurogenesis [58-59]. SE activity increased when neurogenesis was reduced through a pharmacogenetic approach, and a reverse effect was seen when neurogenesis was increased [59]. Strategies that reduced the abnormal integration of newborn neurons also resulted in lower SRS activity in epilepsy models [60, 61]. The present study showed that NSC grafting resulted in the maintenance of a higher level of normal neurogenesis and reduced the abnormal integration of newly born granule cells in the DH even at nine months after SE. Such enduring positive effects on neurogenesis were found to be linked to improved preservation of reelin+ neurons, which are a type of interneurons in the DG guiding the appropriate migration of newly born neurons into the dentate granule cell layer [45]. Thus, positive modulation of neurogenesis response to SE is one of the factors that contributed to reduced SRS activity after NSC grafting.

NSC grafting also modulated another conspicuous abnormal plasticity that occurs in the DG after SE, which is the aberrant sprouting of mossy fibers (i.e., dentate granule cell axons) into the inner molecular layer of the DG seen consistently in various epilepsy models and patients with TLE [62, 63]. Such sprouting increases connectivity between DG granule cells, which could potentially increase DG hyperexcitability. However, the cause-effect link between aberrant mossy fiber sprouting and epileptogenesis has been a matter of debate. Some studies implied that such sprouting engenders a positive feedback seizure-eliciting circuit in the DG [63-68], but other studies suggested that sprouted fibers also excite the surviving inhibitory interneurons, which could lead to better seizure control [69]. Reduced aberrant mossy fiber sprouting at 9 months post-SE in animals receiving grafts could also be due to a slow progression of SRS activity seen in these animals because studies have shown that seizure activity alone can trigger mossy fiber sprouting [70-74]. It could also be due to the better preservation of mossy cells in the DG, resulting in reduced deafferentation of granule cells in animals receiving NSC grafts because the degree of mossy fiber sprouting depends on the extent of mossy cell loss in an epileptic prototype [75]. Regardless of the mechanisms, the results of this present study demonstrated that two distinct types of abnormal plasticity in the DG (abnormal neurogenesis and aberrant mossy fiber sprouting) could be inhibited considerably through early NSC grafting after SE. The net outcome of such effects is a less severe chronic epilepsy with better cognitive function.

Furthermore, NSC grafting resulted in better preservation of host GABA-ergic interneurons in the epileptic hippocampus, which could be discerned from improved survival of subclasses of interneurons expressing NPY and PV in the DG. Such neuroprotective effects could have also contributed to reduced SRS activity in animals receiving NSC grafts because a significant interneuron loss in the DG is linked to hippocampal hyperexcitability as well as the occurrence of SRS. Protection of NPY+ neurons could decrease SRS activity because NPY+ interneurons are involved in inhibiting the activity of hippocampal circuitry by hyperpolarizing excitatory neurons and anticonvulsant activity [76-78]. Likewise, an increased number of PV+ interneurons could decrease seizure activity because deficiency of PV+ interneurons results in increased susceptibility for developing seizures [79], and selective silencing of PV+ interneurons in the hippocampus induces SRS in mice [80]. Collectively, it appears that reduced SRS activity in animals receiving grafts after SE reflects multiple changes mediated by NSC grafts in the epileptic hippocampus. These include a direct contribution of cells (i.e., new GABA-ergic interneurons) and proteins (i.e., the release of GDNF, IGF-1, and FGF-2) having anti-seizure properties, maintenance of higher levels of normal neurogenesis with reduced abnormal integration of newly born neurons, and protection of host GABA-ergic interneurons.

The overall seizure suppression mediated by hippocampal NSC grafts in the chronic phase of epilepsy is somewhat less than that observed with grafting of a pure population of GABA-ergic precursor cells in our previous studies (11, 34). Nonetheless, it is remarkable that grafting of NSCs, resulting in adding a smaller number of GABA-ergic interneurons and a larger fraction of astrocytes into the host hippocampus, is also efficacious for reducing seizures and preserving cognitive function. This finding suggests that apart from the addition of new GABA-ergic interneurons, several other factors that improve host interneurons' survival and function, suppress abnormal plasticity, and enhance normal neurogenesis in the injured hippocampus play roles in diminishing seizures in the chronic phase after SE. These results have implications for clinical translation of cell therapy for epilepsy. Undoubtedly, grafting of a pure population of GABA-ergic interneurons is very attractive because of their ability to greatly suppress seizures [11, 34]. However, the long-term benefits of grafting NSCs capable of differentiating into a smaller percentage of GABA-ergic interneurons and a higher percentage of astrocytes cannot be ignored because such grafting could provide neuroprotection, improve normal neurogenesis, and alleviate astrocyte dysfunction in epilepsy, in addition to suppressing seizures.

### Prospective mechanisms of better cognitive and mood function after NSC grafting

Several mechanisms likely also underlie better cognitive and mood function in animals that received NSC grafts early after SE. Reduced seizure activity is one of the factors contributing to better cognitive and mood function because impaired cognitive and mood functions in chronic epilepsy are linked to seizure activity [81-82], as frequent SRS events could interfere with the synchronized activity of excitatory and inhibitory neurons required for cognitive function or memory formation [83]. NSC grafting promoted higher levels of normal hippocampal neurogenesis with reduced aberrant neurogenesis even at nine months post-SE. Such effects could facilitate better cognitive and mood function because of the involvement of hippocampal neurogenesis in the formation and/or retrieval of different types of memories [84-88] and mood function [89]. Evidence supporting the involvement of normal neurogenesis in mood function includes observations that neurogenesis deficiency leads to increased depressive-like behavior [90] and selective ablation of neurogenesis blocks behavioral responses to chronic antidepressant treatment [91-92]. A study has also shown that the prevention of aberrant hippocampal neurogenesis after SE can also prevent cognitive dysfunction [60]. NSC grafting also resulted in better preservation of NPY+ and PV+ interneurons. Such neuroprotective effects could facilitate better brain function because NPY can directly contribute to better memory and mood function [93-94], or indirectly through maintenance of enhanced hippocampal neurogenesis [95]. Similarly, the involvement of PV+ interneurons in maintaining cognitive function could be seen from memory dysfunction following the selective ablation of PV+ interneurons in the hippocampus [96, 97]. Also, the activity of PV+ interneurons is critical for synchronizing the hippocampal network oscillations involved in data encoding, processing, and storage [98].

Furthermore, NSC grafting possibly resulted in higher levels of neurotrophic factors GDNF, IGF-1, FGF-2, and BDNF in the milieu of the epileptic hippocampus based on the expression of these proteins by significant fractions of graft-derived cells at 9 months post-SE. Increased concentrations of these neurotrophic factors could positively influence cognitive function. The examples supporting this possibility include aged rats exhibiting better cognitive function with the overexpression of GDNF in astrocytes [99], improved cognitive functions seen in an animal model of TLE with the administration of IGF-1 [56], better memory function observed in a model of Alzheimer's disease with FGF-2 gene transfer into the hippocampus, [100], and NSC grafts improving cognitive function in an animal model of Alzheimer's disease through BDNF upregulation [101]. Likewise, studies have also implied that elevated levels of some of these neurotrophic factors could improve mood function. These include studies showing induction of depressive-like behavior following IGF-1 deficiency [102], decreased depressive- and anxiety-like behavior after chronic FGF-2 treatment [103], and improved mood function following peripheral BDNF administration [104]. Thus, it appears that the maintenance of higher levels of normal neurogenesis, diminished abnormal neurogenesis, improved preservation of interneurons, and the release of neurotrophic factors by graft-derived cells underlie improved cognitive and mood function in animals receiving NSC grafts.

### Conclusion

This study demonstrated that intrahippocampal grafting of hippocampal NSCs shortly after SE considerably curbs the progression of epileptogenic processes and the frequency and severity of SRS, which eventually results in a less severe chronic epilepsy with better cognitive and mood function.

## Supplementary Materials

The Supplemenantry data can be found online at: www.aginganddisease.org/EN/10.14336/AD.2020.1020.
