# Supplementary material for: Hippocampal Neural Stem Cell Grafting after Status Epilepticus Alleviates Chronic Epilepsy and Abnormal Plasticity, and Maintains Better Memory and Mood Function
Source: Aging Dis. 2020 Dec 1;11(6):1374–94. doi: 10.14336/AD.2020.1020 (PMC7673840; doi:10.14336/AD.2020.1020)
Supplement: Supplementary file 1 — The Supplemenantry data can be found online at: www.aginganddisease.org/EN/10.14336/AD.2020.1020. [file AD-11-6-1374-s.pdf]

SUPPLEMENTARY DATA

# Hippocampal Neural Stem Cell Grafting after Status Epilepticus Alleviates Chronic Epilepsy and Abnormal Plasticity, and Maintains Better Memory and Mood Function

**Bharathi Hattiangady[12,3,4,#], Ramkumar Kuruba[3,4,#], Bing Shuai[1,2,3,4], Remedios Grier[3,4], Ashok K. Shetty[12,3,4*]**

© 2020. Hattiangady B et al. Published online at http://www.aginganddisease.org/EN/10.14336/AD.2020.1020

# SUPPLEMENTARY DATA

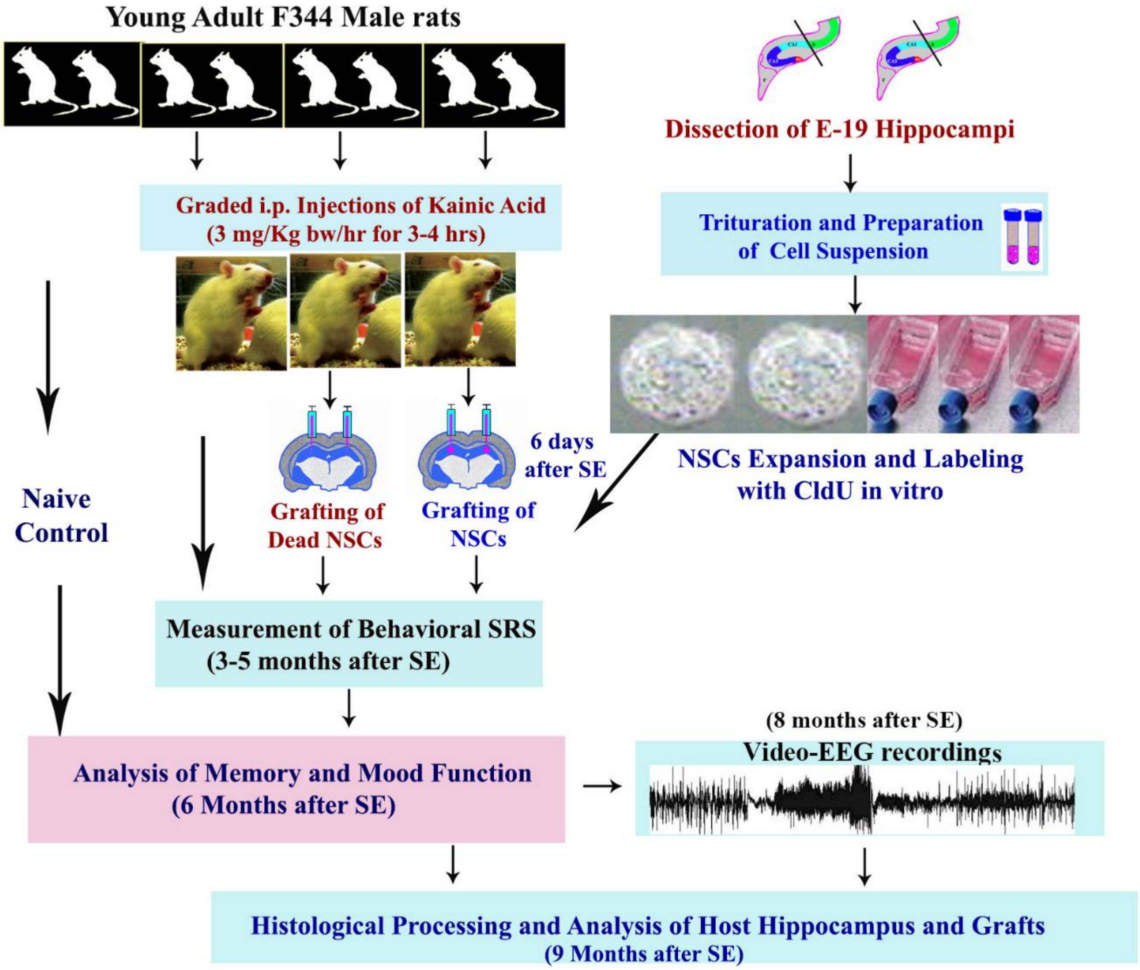


**Supplementary Figure 1. The figure depicts an overview of the experimental design.** The right side of the figure shows the dissection of embryonic day 19 (E19) hippocampi, trituration of hippocampal tissues, preparation of single-cell suspension, expansion of neural stem cells (NSCs) as neurospheres, and labeling of NSCs with 5'-Chloro-2'-deoxyuridine (CldU) in vitro. Neurospheres were triturated into a cell suspension and treated with brain-derived neurotrophic factor (BDNF) before grafting into the hippocampus. The figure's left side depicts the induction of SE in F344 male rats by graded intraperitoneal (i.p.) injections of excitotoxin kainic acid. Grafting of NSCs (or dead NSCs) was performed bilaterally into hippocampi at six days after post-SE. The frequency, intensity, and duration of behavioral spontaneous recurrent seizures (SRS) were measured from all groups via direct observations at 3-5 months post-SE. The animals were examined for memory and mood function six months after SE. A continuous video-encephalographic (video-EEG) recordings were done eight months after SE. Animals were euthanized nine months after SE for histological analyses of grafts and the host hippocampus.

© 2020. Hattiangady B et al. Published online at http://www.aginganddisease.org/EN/10.14336/AD.2020.1020
